# Supplementary material for: Novel Micellar Formulation of Silymarin (Milk Thistle) with Enhanced Bioavailability in a Double-Blind, Randomized, Crossover Human Trial
Source: Pharmaceutics. 2025 Jul 4;17(7):880. doi: 10.3390/pharmaceutics17070880 (PMC12298458; doi:10.3390/pharmaceutics17070880)
Supplement: Supplementary file 1 [file pharmaceutics-17-00880-s001.zip › pharmaceutics-3716335-supplementary.pdf]

Supplementary Material S1: Adverse Event Monitoring Questionnaire

To assess the safety and tolerability of the study formulations, participants were asked to complete a structured adverse event (AE) questionnaire 24 hours after each treatment period. The form was designed to capture both expected and unexpected symptoms, graded by severity using a standardized 5-point scale:

Severity Score	Definition
0	No symptoms
1	Mild (noticeable but easily tolerated; no intervention needed)
2	Moderate (some interference with daily activity; no medical treatment required)
3	Severe (marked interference with daily life; may require medical intervention)
4	Life-threatening or disabling

Participants rated the severity of the following predefined symptoms:

Symptom	No symptoms	Mild	Moderate	Severe	Life-threatening
Bloating (feeling of fullness or pressure)					
Constipation					
Diarrhea					
Heartburn					
Abdominal pain/cramping/knotted sensation					
Rash					
Nausea					
Dizziness					
Blurred vision					

In addition to the severity ratings, participants responded to the following supplemental questions:

- How long did the symptoms last?
- Did the adverse events affect your daily activities or quality of life?
- Did you require any additional medication or treatment?
- Did you inform your healthcare provider about the symptoms?
- Were the symptoms tolerable, or did they significantly impact your ability to continue the study?
- Did you experience any other symptoms not listed above? Please describe.

Completed questionnaires were reviewed by study staff, and any symptom rated ≥2 was flagged for follow-up.
